# Effects of Implementing a Brief Family Nursing Intervention With Hospitalized Oncology Patients and Their Families in Germany: A Quasi-Experimental Study

**DOI:** 10.1177/1074840720967022

**Published:** 2020-12-07

**Authors:** Manuel Zimansky, Lukasz Stasielowicz, Inga Franke, Hartmut Remmers, Heiko Friedel, Jens Atzpodien

**Affiliations:** 1 Osnabrück University, Germany; 2 Paracelsus-Wittekindklinik Bad Essen, Germany; 3 Gesellschaft für Gesundheitsmanagement mbH, Essen, Germany; 4 Franziskus-Hospital Harderberg, Georgsmarienhütte, Germany

**Keywords:** Calgary Family Assessment and Intervention Models, family nursing, inpatient oncology, knowledge transfer, Germany

## Abstract

Family nursing, based on the Calgary Family and Intervention Models, was implemented in a German oncological inpatient unit to promote effective family functioning in the context of cancer care. The objective of this study was to investigate the effects of implementing family nursing care on several psychological and physical outcomes of patients and their family members. A quasi-experimental study with 214 patients with a cancer diagnosis and 122 family members was conducted. Findings indicate that the superiority of family nursing, when compared to traditional care, could not be confirmed with respect to patients’ outcomes (psychological burden, social support, satisfaction with care) and family members’ outcomes (psychological burden, physical complaints, satisfaction with care). Various factors, such as country-specific structures and challenges in implementing family nursing care on an inpatient unit, may have contributed to these findings. Further replication attempts in similar settings in other countries are needed to shed light on the factors impairing or promoting the implementation of family nursing in practice settings.

A cancer diagnosis affects not only those individuals who receive the diagnosis, but also their close family members, and their broader social context. A life-threatening cancer illness can have such an impact on the family that it leads to destabilization of the family system and family members often experience considerable physical, social, and emotional burdens (Stenberg et al., 2010). The whole family is in need of support and advice from health care professionals to alleviate illness suffering and to strengthen their ability to cope with the illness (Dieperink et al., 2018; Given et al., 2012). To provide support for families facing cancer, numerous psychosocially oriented family interventions, tailored to the population affected by cancer, have been developed. The effectiveness of these interventions has been reported in a large number of studies (Fletcher et al., 2012). Research findings indicate that psychosocial interventions delivered to cancer patients and their relatives have small to medium positive effects on multiple outcomes (Badr & Krebs, 2013; Baik & Adams, 2011; Hopkinson et al., 2012; Northouse et al., 2010). Medium effect sizes were found, in particular, for supportive-educational interventions focusing on a wide range of issues including, for example, family involvement, symptom management, and coping skills (Waldron et al., 2013).

Besides the growth of research evidence about family interventions, noticeable theoretical progress has been made in the field of family nursing and several models for nursing practice with families have been developed worldwide over the past 30 years (Denham, 2003; Friedemann, 1995; Gottlieb, 2013; Hohashi & Honda, 2011; Wright & Bell, 2009; Wright & Leahey, 2013).

The focus of this study was to examine the effects of family intervention informed by Family Systems Nursing models. The uniqueness of Family Systems Nursing is that it is focused on the interaction, reciprocity, and relationships between multiple systems levels, rather than one particular level, that is, the illness, the ill person, the family, the health care provider, and larger levels (e.g., health care system, culture). The goal is to promote family health and well-being by directing nursing toward both health promotion and relief from illness suffering (Bell, 2009).

Well-known Family Systems Nursing models include the Calgary Family Assessment Model (CFAM) and the Calgary Family Intervention Model (CFIM) developed in Canada by Wright and Leahey (2013) (Shajani & Snell, 2019). The CFAM enables nurses to comprehensively assess current family strengths, resources, problems, and illness suffering through targeted questions that assess family structure, development, and function. To assess the family’s structure and interactions with their environment, two structural assessment tools, the genogram and ecomap, are typically used (Rempel et al., 2007; Wright & Leahey, 2013). The CFIM provides an organizing framework for the nurse-family relationship and for a therapeutic conversations offered by nurses using specific family nursing interventions (e.g., interventive questions, commendations) that target the systems level that offers the greatest opportunities for family health and healing (Bell & Wright, 2015). CFAM and CFIM can be used by nurses in order to help families address their illness concerns as well as promote family health/well-being and effective individual and family functioning (Robinson & Wright, 1995; Wright & Leahey, 2013).

The CFAM and CFIM models have been introduced and studied in several countries (Leahey & Wright, 2016). Qualitative studies have been carried out in Canadian pediatric hospitals (LeGrow & Rossen, 2005; Martinez et al., 2007), Canadian medical-surgical units (Leahey et al., 1995), a Canadian psychiatric unit (Goudreau et al., 2006), heart failure outpatient clinics in Denmark (Voltelen et al., 2016), and inpatient and outpatient care in Iceland (Svavarsdottir et al., 2015). Recently, research has been conducted in Iceland to examine the impact of implementing a family-oriented approach in specialized palliative home care (Petursdottir et al., 2019; Petursdottir & Svavarsdottir, 2019).

In Switzerland, data about the evaluation of the implementation process of the Calgary models in oncology have focused on both anecdotal evidence from a family nursing implementation project in an inpatient oncology unit in Switzerland (Preusse-Bleuler, 2009) and more recently, in a family nursing implementation project at Zurich University Hospital (Naef, Kaepelli, et al., 2020; Naef, Kläusler-Troxler, et al., 2020). Recommendations include greater attention to the implementation strategies used and the dose and frequency of the educational programs offered to nurses to grow and support their ability to provide quality family nursing care.

While initial attempts to implement family nursing based on the Calgary models have been made in selected countries over the past several years, the respective concepts and necessary structures for family nursing have been lacking in German oncology units (Pinkert et al., 2011). This study reports a first attempt to implement and evaluate family nursing based on the Calgary models in a German oncological inpatient unit located in Lower Saxony, Germany, from 2014 to 2017.

## Aims of the Study

In addition to the introduction of family nursing in an oncology unit in Germany, the objectives of this family nursing implementation project were as follows: (a) to systematically record, evaluate, and if necessary to modify the implementation process of family nursing (formative evaluation), and (b) to investigate the effects of family nursing on the patients and their families (summative evaluation). In short, we wanted to test whether family nursing based on the Calgary models could be easily adopted in a German oncological unit and examine its purported benefit.

The results of the formative evaluation are available elsewhere (Zimansky et al., 2018). In the present article, the effects of family nursing on several patient and family outcomes are reported: stress, mood, anxiety, physical complaints, satisfaction, and social support. The choice of the outcomes was motivated by the assumptions of the Calgary models, which link family nursing to improved family health/well-being and effective family functioning in the cognitive, affective, and behavioral domains (Wright & Leahey, 2013).

As part of the summative evaluation, it was expected that after the introduction of family nursing:

1. Patients: (a) would experience less psychological burden (stress, mood), (b) would receive more social support from their relatives, and (c) would be more satisfied at the end of the hospital stay than patients who were hospitalized before the introduction of family nursing;
2. Family members: (a) would experience less psychological burden (stress, anxiety), (b) would report fewer physical complaints, and (c) would report being more satisfied at the end of the hospital stay of their affected relatives than family members whose affected relatives who were hospitalized before the introduction of family nursing.

## Method

### Design

The effects of family nursing, based on the Calgary models, on patients and family members in an oncological unit in Germany were analyzed using a quasi-experimental design. Specifically, patients who were admitted to an oncological unit (and their families) before the implementation of family nursing (June 2014—May 2015) were regarded as the control group. Patients and family members who received family nursing care following an educational program for nurses and the implementation of family nursing in the oncological unit (December 2015—January 2017) were regarded as the intervention group.

### Sample

The final sample included only patients and their relatives who were older than 18 years and were able to complete the questionnaires. In addition, all patients were in at least the second treatment phase and their family members agreed to participate in the study. All family members were described by the patient as “close relatives” and were aware of the patient’s diagnosis.

### Data Collection

Data were collected from both control groups and intervention groups in the oncological inpatient unit, after obtaining ethical approval from the Research Ethics Committee of Osnabrück University. The recruitment of patients and their family members took place one day after the patient’s admission. Patients and family members who met the inclusion criteria were verbally informed by a research team member about the project and were asked to participate in the study. They were also given an information letter, in which the investigation was briefly described. With the assurance that their participation was voluntary, assurance of anonymity, and the right to withdraw from the study at any time, the patients and family members declared their willingness to participate individually by signing a consent form. Both questionnaires were handed out to the participating persons with the request to complete the admission questionnaire immediately and the second questionnaire on the day of discharge. As a rule, a research team member collected the questionnaires. In addition, there was a possibility of giving the completed questionnaire in a sealed envelope to a nurse or sending it in a pre-postmarked envelope to the clinic following discharge from the hospital.

### Description of the Intervention: Family Nursing Implementation

Under the supervision of a project management team, consisting of two researchers from Osnabrück University and two nurses from the oncological unit, family nursing was implemented on the oncological unit in a 6-month period from June to November 2015. All 25 nurses of the 32-bed oncological unit were educated by a family therapist in the basics of family nursing using a five-module course consisting of 3 hr of lecture per module. The first three modules focused on the theoretical foundations of systemic intervention and served as a means of imparting a context-sensitive case-comprehension and an empathic, appreciatively communicative attitude toward families. Genograms, ecomaps, and an interprofessional team intervention were discussed and applied by the nursing staff. Within the framework of the last two modules, the entire nursing team was educated in the use of therapeutic conversations in the family interview sessions. Various conversation scenarios were tested in small groups. Behavior in conflict situations as well as the avoidance of frequent errors in family nursing were also discussed. In addition to the educational course, all nurses of the oncological unit were given 12 clinical sessions lasting 1.5 hr each by a family therapist about conducting family assessments, family interviews, and family-related team meetings. The other non-nurse treatment team members (physicians, psycho-oncologists, and social workers) received a 3-hr short course which focused on the use of genograms, ecomaps, and the interprofessional team intervention.

The family intervention implementation process consisted of two group interviews with nurses, five interviews with other members of the treatment team, and observations of four family assessments, family interviews, and family-related team meetings. Based on the assessment results, a continuous adjustment of the implementation process was carried out. The following approach to family nursing, tailored to the specific requirements of the German oncology unit, was considered practical by the nurses, physicians, psycho-oncologists, and social workers of the oncology unit.

During the family assessments, each lasting 5 to 10 min, nurses asked all patients and their family members at the beginning of the hospital stay about their specific family structure, health-related burdens, and experiences as well as family resources and problems, depending on the focus of the conversation. Family relationships were graphically depicted through genograms and ecomaps. Family interviews were usually conducted once with all patients and their family members during their stay and lasted between 5 and 30 min. In the interviews available resources in the social environment as well as limitations and support needs identified in the previous family assessment were discussed with the family in a solution-oriented manner. Existing or possible future symptoms and impairments were explored and a conversation about possibilities of dealing with them was encouraged by using circular questions. In addition, uncertainties related to the discharge as well as the need for post-discharge care were discussed. Attention was drawn to the availability of support services of social workers and psycho-oncologists.

In addition to the implementation of the Calgary models and as an integral part of the work in the oncology unit, family-related team meetings were institutionalized to strengthen a collaborative relationship within the treatment team and between treatment team members and the family. For these meetings, an interprofessional team intervention was developed, tailored to the specific working culture of the treatment team. During the interprofessional team meetings, which were organized whenever needed, the responsible nurses presented the situation of families with complex problems. In a subsequent process of alternating directed and non-directed communication, the attending nurses, physicians, psycho-oncologists, and social workers reflected the problems of the family and formulated solutions that could later be proposed to the family.

### Measures

To investigate the effects of the family nursing interventions on the patients and their families, several outcomes were assessed. Psychological burden was identified for both patients (stress, mood) and family members (stress, anxiety). In addition, physical burden (i.e., headaches, fatigue) of family members were assessed. The effects of the interventions on family functioning, especially in the affective and behavioral domains, were determined by assessing patients’ und family members’ satisfaction, and by measuring family members’ capacity for provision of social support. The outcomes were assessed on two measurement occasions for both control groups and intervention groups. Table 1 contains a summary of the crucial variables assessed in the present study. In the following paragraphs, we describe the instruments used to assess the outcomes.

**Table 1.** Investigation Plan for Patients and Family Members.

Group	One day after admission (t_0_)	Day of discharge (t_1_)
Patients (*n* = 214)	Psychological burden: • Stress: Questionnaire on Stress in Cancer Patients • Mood: Adjective Mood Scale—Revised version	Psychological burden: • Stress: Questionnaire on Stress in Cancer Patients • Mood: Adjective Mood Scale—Revised version Family’s capacity for provision of social support: • Berlin Social Support Scales Satisfaction: • Satisfaction questionnaire Sample information: • Sociodemographic questionnaire
Family members (*n* = 122)	Psychological burden: • Stress: Perceived Stress Questionnaire • Anxiety: State-Anxiety part of the State-Trait Personality Inventory Physical complaints: • Giessen Subjective Complaints List	Psychological burden: • Stress: Perceived Stress Questionnaire • Anxiety: State-Anxiety part of the State-Trait Personality inventory Physical complaints: • Giessen Subjective Complaints List Satisfaction: • Satisfaction questionnaire Sample information: • Sociodemographic questionnaire

#### Patients

The baseline values of the variables that were assessed twice were measured 1 day after admission (t_0_) for the first time, both in the control group and in the intervention group. The second survey took place on the day of discharge (t_1_) and included some additional questions (i.e., related to satisfaction with the care received). Stay duration varied between the patients, but all patients from the inpatient group stayed at the hospital for at least 3 days. Patients’ subjective stress experience and mood were used as indicators of psychological burden and gauged using two standardized and established instruments. Specifically, the Questionnaire on Stress in Cancer Patients (QSC-R23), in its revised form (Herschbach et al., 2004), and the Adjective Mood Scale (AMS-R) in the revised version (von Zerssen & Petermann, 2011). The former consists of 23 six-point items (0 = *does not apply to me*, 1 = *applies to me, is only a slight problem*, 5 = *applies to me, is a very big problem*) that pertain to everyday stress, that is, “I often feel tired and weak.” The AMS-R was added because it has a higher rate of change sensitivity in comparison to the QSC-R23 as it measures the current mood. Thus, it is more suitable for testing the immediate effect of family nursing on the patients between admission and discharge. AMS-R consists of 24 pairs of antonymic adjectives (i.e., glad vs melancholic) and people are asked to indicate whether the first, second, or none of the adjectives applies to them. Both instruments have been examined for their psychometric properties. In the present study, the QSC-R23 reliability (Cronbach’s alpha) was very good on both admission and discharge measurement occasion (α = .92 for both). Similarly, the AMS-R had good reliability in our sample (α = .94 on both admission and discharge).

To investigate the patient’s experience of the family’s capacity for provision of social support the *Berlin Social Support Scales* (BSSS) were submitted on the day of discharge (t_1_). The BSSS (α = .90) measure both cognitive and behavioral aspects of social support (Schulz & Schwarzer, 2003, 2004). Twelve four-point items (1 = *not at all*, 4 = *very much so*) were used, which include items like “This person was there when I needed him or her.”

Patients’ satisfaction, especially with care and the oncological unit in general, was recorded on the day of discharge (t_1_). The self-developed *satisfaction questionnaire* included items on which the patients stated their level of satisfaction with the support from the nursing staff, the inclusion of their relatives in care, the perception of the family situation and the overall hospital stay. Fifteen five-point items (0 = *was not important for me*, 1 = *fully met*, 4 = *not at all met*) pertaining to satisfaction with specific aspects (e.g., “Being consoled”) as well as three five-point Likert-type items (1 = *fully satisfied*, 5 = *completely dissatisfied*), which were broader (i.e., “How satisfied were you in total with the support from the nursing staff?”), were used in the satisfaction questionnaire. Due to different response formats, the respective items were z-standardized before computing scale mean and scale reliability (α = .93).

A sociodemographic questionnaire was submitted to the patients on the day of discharge (t_1_) to collect descriptive sample information which could serve as covariates for the covariance analyses. Questions asked pertained to the person’s characteristics (age, sex, education), family (partnership, important persons, residential situation, number of minor children), illness (initial diagnosis, type of illness, occasion of current treatment), and therapy (chemotherapy, radiotherapy).

In addition, to account for potentially confounding variables, the patient’s diagnoses (metastases, number of International Classification of Diseases 10th Revision [ICD-10] diagnoses) and patient’s level of functioning in terms of their ability to care for themselves, daily activity, and physical ability (Eastern Cooperative Oncology Group Score [ECOG] score) were recorded. Furthermore, type, frequency, and dosage of analgesics, antiemetics, antidepressants, and sleep-promoting drugs were recorded to control for medication effects. These objective health data were collected after the patient was discharged.

#### Family members

The timing of data collection in the control group as well as in the intervention group of the family members was synchronized with the survey among the patients. This means the baseline values were also measured one day after the patient’s admission (t_0_) and the second survey took place on the day of discharge (t_1_). Since no specific German instruments were available to assess the psychological burden of family members of persons affected by a somatic disease, instruments developed for use in a healthy person were utilized. The psychological burden of the family members was recorded on the basis of the subjective stress experience as well as the momentary state of anxiety. Specifically, the Perceived Stress Questionnaire (PSQ) in the German version (Fliege et al., 2001; Levenstein et al., 1993) and the State-Anxiety part of the German version of the State-Trait Personality Inventory (STPI State) were used (Schwarzer, 1981; Spielberg, 1995). PSQ consists of 30 four-point items (1 = *almost never*, 4 = *usually*) and includes items like “You feel rested.” In the current study, the scale reliability was very good (α = .97 on both admission and discharge). State-anxiety was measured with 10 four-point items (1 = *not at all*, 4 = *very much so*) pertaining to state rather than trait aspects, e.g., “I am tense.” Reliability was good in the present sample (α = .84 on both admission and discharge).

The short form of the Giessen Subjective Complaints List (GSCL-24) was used to assess the physical complaints of the relatives (Brähler et al., 2008; Schlagenhauf, 2003; Wilz et al., 2005). GSCL-24 has 24 five-point items (0 = *no complaints*, 4 = *strong complaints*) referring to physical symptoms (i.e., headaches, fatigue). In the current sample, the reliability was also very good on both measurement occasions (α = .94).

In addition, the satisfaction of family members with the interaction with nursing staff was recorded on the day of discharge (t_1_). The basis for the satisfaction questionnaire formed the results of the previous study, where the most important needs of relatives which could be met by nurses were identified (Pinkert et al., 2011). The degree of satisfaction of the relatives with the interaction with nursing staff was recorded by asking whether the respective needs, for example, “to always have a contact person on the unit” or “to be treated on a joint and equal basis,” were fulfilled. Fulfillment of those 20 needs was rated on a five-point scale (0 = *was not important for me*, 1 = *fully met*, 4 = *not at all met*). The satisfaction questionnaire had very good reliability in the present sample (α = .99 for both admission and discharge).

A sociodemographic questionnaire was used to collect anonymous personal data from the relatives on the day of discharge (t_1_). Individual characteristics (age, sex, education) and relationship with the affected person (e.g., degree of relationship, residential situation) were assessed.

### Data Analysis

The main statistical analyses were conducted with SPSS 24. To test the hypothesis that the patients from the intervention group experienced less psychological burden than people in the control group (AMS-R, QSC-R23) we conducted two covariance analyses (ANCOVA) with different sets of covariates. Covariates that were included in at least one of the two models were admission values on the respective psychological burden scale, year of birth, and number of ICD-10 diagnoses. Other hypotheses concerning patients (social support, satisfaction) were tested by comparing group means of the intervention group and control group using a *t*-test, because no relevant covariates could be identified. In addition, we conducted dropout analyses to check whether people who participated in the whole study differed in some characteristics (metastases, number of ICD-10 diagnoses, ECOG score, and use of drugs) from people who only delivered admission data or no data at all. The analyses were conducted separately for intervention group and control group. Based on the results, we decided to consider health-related variables as covariates in our main analyses together with sociodemographic variables. Variables that were related to the respective outcome variable were included in the relevant models when testing the hypotheses.

For family members, we used ANCOVA (with year of birth and education as covariates) to compare the psychological burden (PSQ, STPI-State), physical complaints (GSCL-24), and satisfaction at the discharge between the intervention group and control group. To account for confounding variables we checked whether the sociodemographic variables and admission values of the outcome variable were related to the outcome variable at the discharge.

## Results

In accordance with the order of our hypotheses, we first report the results pertaining to patients, followed by the results concerning family members. The results also report information about sample characteristics (see Tables 2 and 3) and comparisons between the intervention group and the control group (i.e., dropout rate, health differences between groups).

**Table 2.** Sample Characteristics (Patients: *N* = 214).

Variable	*Mdn.*/frequency	
	Intervention group	Control group
Sex		
Men	83	43
Women	42	45
Year of birth	1951	1951
Education		
No education	0	1
Secondary education	112	80
College degree	13	7
Civil status		
Married	87	71
In a relationship	6	3
Single	9	2
Divorced	10	2
Widowed	9	10
Number of children	2	2
Dwelling		
House	86	65
Flat	29	19
Assisted living	2	1
Other	2	0
Metastases		
No	39	21
Yes	83	56
ECOG score (performance status)	1	1
Drugs	2	2
Number of ICD-Diagnoses	5	6

*Note.* ICD = International Classification of Diseases 10th Revision; ECOG score = Eastern Cooperative Oncology Group Score; Drugs = use of painkillers, antiemetics, sleeping pills, antidepressants, anxiolytic (composite score, max = 5).

**Table 3.** Sample Characteristics (Family Members: *N* = 122).

Variable	*Mdn.*/frequency	
	Intervention group	Control group
Sex		
Men	14	31
Women	40	37
Year of birth	1958.5	1955
Education		
Secondary education	43	52
College degree	11	15

**Table 4.** Sample Characteristics (Family Members—Outpatient Group only, *N* = 100).

Variable	*Mdn.*/frequency
Sex	
Men	54
Women	45
Year of birth	1958.5
Education	
Secondary education	71
College degree	27

*Note.* For results pertaining to this group, see Note 1.

### Sample Characteristics

#### Patients

In total, 516 patients met the inclusion criteria and they were approached and asked to participate. Table 2 contains a summary of sample characteristics: 214 (41.47%) patients completed the scales. Considering the severity of the disease, it was not surprising that not all patients completed the scales on both measurement occasions (158 patients on both measurement occasions, and 56 on admission only). Of those who participated and reported relevant data 59.2% were men. More than half of the sample was at least 60 years old (*Mdn* = 1951). All patients were born between 1924 and 1988.

In the intervention group, no statistically significant differences were found between patients who completed all questionnaires and patients who did not want to participate (all *p*s ≥ .140). However, two differences between patients who participated in the whole study and patients who delivered only admission data were identified. Specifically, patients from the final sample had less ICD-10 diagnoses, *t*(120) = 3.45, *p* = .001, *d* = -0.67, and less metastases in respiratory or digestives organs (ϕ = -.20, *p* = .027, *N* = 125). All other differences were not statistically significant (all *p*s ≥ .063). In the control group, only one significant difference was found between the final sample and people who did not want to participate in the study at all. The latter had a worse ECOG score, *t*(146) = 2.26, *p* = .025, *d* = -0.37. However, there were no further differences between the two groups (all *p*s ≥ .246). There were also no differences between patients who participated in the whole study and patients who only delivered admission data (all *p*s ≥ .280). Summing up, those findings do not lend support to the explanation that the high dropout rate (58.53%) was caused by severity of the disease.

Some differences in the final sample between patients from the intervention group and the control group could be found with respect to their health state (metastases, number of ICD-10 diagnoses, ECOG score, and drugs). Specifically, a greater proportion of patients in the intervention group than in the control group had metastases in lymph nodes (*ϕ* = .14, *p* = .049, *N* = 210) and had no metastases in respiratory or digestive organs (*ϕ* = -.18, *p* = .010, *N* = 210). Moreover, patients from the intervention group took more drugs, *t*(161) = 2.76, *p* = .006, *d* = 0.45, than people in the control group.

#### Family members

With respect to the sociodemographic variables of family members, the family members were, on average, somewhat younger than patients, with a median year of birth of 1957. Most of the family members were female and the disproportion between men and women was somewhat larger in the intervention group. Furthermore, less than a quarter of the sample had a college degree. Table 3 includes a summary of the relatives’ characteristics (*N* = 122).

### Findings: Comparison of the Intervention Group and the Control Group

#### Patients

Contrary to our expectations, stress differences between the patients in the intervention group and patients in the control group were statistically insignificant, *F*(1, 149) = 0.94, *p* = .335, $\eta_{p}^{2}$ = .01, after accounting for covariates (year of birth and admission values). Moreover, there were no differences at the time of discharge with respect to the mood, *F*(1, 113) = 3.30, *p* = .072, $\eta_{p}^{2}$ = .028, after controlling for mood at admission and number of ICD-10 diagnoses. Similarly, patients from the intervention group did not experience better social support from relatives, according to the BSSS scores, *t*(151) = 0.49, *p* = .313, *d* = 0.08. Furthermore, patients in the intervention group were somewhat less satisfied than people in the control group at the time of discharge but the difference was not statistically significant, *t*(125) = -1.52, *p* = .066, *d* = -0.27.

#### Family members

There were no differences between the intervention group and the control group with regard to stress, after accounting for education and perceived stress at admission, *F*(1, 85) = 0.03, *p* = .869, $\eta_{p}^{2}$ = .00. The two groups also did not differ with respect to anxiety levels, when we adjusted for education and anxiety at admission, *F*(1, 82) = 0.07, *p* = .787, $\eta_{p}^{2}$ = .00. With respect to family members’ physical complaints, no differences were found between the intervention group and the control group at the time of discharge as evidenced by the ANCOVA result after accounting for physical complaints at admission, *F*(1, 85) = 2.06, *p* = .155, $\eta_{p}^{2}$ = .02. Similarly, family members of patients from the intervention group did not report better satisfaction levels than family members of people from the control group after accounting for year of birth and education, *F*(1, 45) = 0.15, *p* = .697, $\eta_{p}^{2}$ = .00. However, it has to be noted that many relatives indicated that certain care aspects were not at all important for them. Thus, they did not indicate their satisfaction level on several items, which rendered it impossible to consider data of those relatives in the satisfaction analysis.^1^

## Discussion

The effectiveness of family nursing based on the CFAM and CFIM on patients and their relatives in oncology was evaluated for the first time in Germany with this study. Advantages of family nursing could not be shown with respect to patients’ outcomes (psychological burden, received social support, satisfaction) and family members’ outcomes (psychological burden, physical complaints, satisfaction).

In other studies, it has also been shown that family nursing does not necessarily yield benefits with respect to certain outcomes. Although a positive effect of the short therapeutic conversation intervention on parents’ perceived cognitive support at an Icelandic Children’s Hospital could be demonstrated by using an experimental research design, a significantly higher perceived emotional support after the intervention could not be found in the intervention group (Svavarsdottir et al., 2012). A somewhat different pattern was found in a study conducted at a psychiatric division at a University Hospital in Iceland. Whereas family members in the intervention group reported significantly higher cognitive and emotional support no benefits were found for patients. Specifically, patients from the intervention group did not report higher cognitive or emotional support nor did they report higher family support (Sveinbjarnardottir et al., 2013). Taken together those findings and the results of our study indicate that the evidence of superiority of family nursing in hospital settings is mixed at best.

Several factors, like country-specific structures (e.g., high nurse-to-patient ratios) and implementation problems may have contributed to our unexpected findings. The results of our complementary formative evaluation study, in which the implementation process was systematically reviewed, indicate that family nursing based on the CFAM and CFIM could only be implemented in an adjusted form in the oncological unit, tailored to existing conditions in Germany (Zimansky et al., 2018). The complete implementation was impeded by the lack of professional consulting competencies of the nursing staff, the system of nursing care delivery, and lack of time. The deficit in professional consulting competences of the nurses may be due to the lack of developed consulting concepts in German nursing care (Zwicker-Pelzer et al., 2011). In family interviews conducted by the nurses in the oncological unit, circular questions were used only sporadically and detached from a methodically well-trained systemic conversation. Furthermore, not all nurses could fully develop a family-centered attitude during the implementation process. They had difficulties in conceptualizing multiple systems levels simultaneously and selecting appropriate interventions. Overall, a shift toward family nursing was not completely achieved in the oncological unit with respect to work culture and mind-set.

It is possible that the short duration of the nurses’ preparation for this study contributed to the unexpected findings, although our study was aligned to other investigations. To illustrate, compared to the 2-day education in our study, in other studies the educational family nursing course for nurses was completed in 4 hr (Martinez et al., 2007), 1 day (LeGrow & Rossen, 2005; Svavarsdottir et al., 2015; Sveinbjarnardottir et al., 2013), 4 days (Goudreau et al., 2006), and 8 days (Preusse-Bleuler, 2009). The education period was considered to be adequate in almost all of those studies. Only Martinez and colleagues (2007) stated that further education was needed. Thus, the educational course duration in our study did not deviate from other interventions and should have enabled successful implementation of family nursing. It is also possible that the implementation phase of 6 months was too short and a longer implementation period with continuous education would have contributed to a more successful implementation of family nursing. Bell and Wright (2011) point out that acquiring clinical excellence requires “deliberate practice” (Gladwell, 2008) in family nursing and many clinical lessons over time. Östlund et al. (2015) recommend continuous learning of theory and practice of family nursing over a long period, because more time is needed for the nurses to internalize core components of family nursing. Duhamel (2017) stresses the importance of mentors or coaches in the clinical workplace during this period, who can serve as role models for all members of the treatment team when implementing family nursing.

To further explore the reasons for mixed findings regarding the effectiveness of family nursing based on the CFAM and CFIM, more research is needed. Further investigations could shed more light on the factors limiting the effectiveness of such interventions (i.e., outcomes assessed, type of hospital unit, country). Such findings would make it easier for decision-makers to choose appropriate implementation strategies. However, future studies could also explore the role of factors which have not been examined before, that is, the influence of spirituality in the context of family nursing. Although nurses explore families’ spiritual beliefs and practices, spirituality plays only a marginal role in Calgary models. Its potential importance has been acknowledged in other models, that is, the Trinity Model (Wright, 2017), and specific guidelines related to spiritual interventions do exist (Tanyi, 2006). It has been shown that spiritual interventions may contribute to family resilience (Black & Lobo, 2008) and that highly spiritual cancer patients report better health than less spiritual people (Jim et al., 2015). Thus, it may be an important covariate in studies based on subjective outcomes like those in the present study (i.e., perceived stress, mood). Another variable, which may be relevant in the context of family nursing, is adaptive performance or adaptive expertise (Baard et al., 2014; Jundt et al., 2015; Maynard et al., 2015). People differ in their capacity to adapt to changes. Implementation of family nursing may be challenging to some nurses, because such a massive intervention requires breaking usual routines and practices (e.g., communication rules). It seems to be crucial to ensure that individual nurses and teams can adapt to changing circumstances, that is, by facilitating adaptive performance (Joung et al., 2006). Conversely, the success of a family nursing intervention may depend on individual nurses’ or teams’ adaptability. Thus, it may be fruitful to consider this aspect in future studies.

### Limitations

Notwithstanding the robustness of our findings, some limitations have to be acknowledged. One could argue that the lack of the evidence for the effectiveness of family nursing in our study was due to the short stay at the hospital (3 days for a few patients). It may be a valid point in the case of stress, as it may require some time to reduce the stress level, but even within this short period a significant stress reduction could be observed in the control group as opposed to the intervention group. Furthermore, a more change sensitive measure (mood) was also included in the current study, but, again, an improvement could only be seen in the control group. Importantly, all instruments had good or very good reliability (all αs ≥ .84). Thus, it is unlikely that the supposed superiority of family nursing could not be shown due to the short stay at the hospital or due to the type of instruments utilized in the present study. Nevertheless, it cannot be ruled out that family nursing had an impact on other outcomes. The assessed outcomes (burden, social support, and satisfaction) can be derived from the CFIM and CFAM, because they refer to family health and family functioning outcomes. However, it can be argued that one should not restrict the evaluation to individual outcomes of different family members but also consider the influence on the relationships within the family. To illustrate, another research group used the Iceland Expressive Family Functioning Questionnaire (ICE-EFFQ) for measuring family functioning and the Iceland-Family Members’ Perceived Support Questionnaire (ICE-FPSQ) to evaluate the families’ perceived support from nurses and other professionals who provide family interventions (Sveinbjarnardottir et al., 2012a, 2012b). Gilliss and colleagues (2019) point out, that the majority of studies on family involvement in adult chronic disease care only show effectiveness in changing the behavior or health status of the family when the coping ability or partnership between family members was the focus of the intervention. In the current study, only the family’s capacity for provision of social support was assessed as an outcome explicitly involving other family members.

Another potential limitation of the study pertains to the high drop-out rate. However, high attrition rates are not unusual in health-related settings (Galea & Tracy, 2007). Furthermore, we systematically compared patients, who did not want to participate in the study with patients, who delivered partial or complete data and found only negligible differences with respect to objective health data (i.e., metastases). Thus, in spite of high drop-out rate our sample included people with severe health problems.

## Conclusion

In contrast to some previous studies, we could not confirm that patients and their relatives benefit from family nursing based on the CFAM and CFIM when decision-makers are conducting a cost-benefit analysis. This is to be countered by the fact that other researchers have found positive effects of family nursing based on the CFAM and CFIM, at least in other settings (i.e., pediatrics, psychiatry) and that in our study country-specific structures as well as implementation problems may have led to unexpected findings. We conclude that even before the introduction of family nursing in German oncology inpatient units, certain basic requirements should be met to enable finding of the effects. As the introduction of family nursing approaches, the nurses should already have been working according to the principles of primary nursing. A high proportion of the nursing staff should have basic professional consulting competences, which serve as a basis for the acquisition of general family nursing competencies. During the implementation process, a gradual introduction of family nursing based on the evolving competencies of the nurses over a longer period seems recommended, because more time is needed for the nurses to internalize core components of family nursing and to develop a family-centered attitude.

We look forward to further replication attempts, that is, studies conducted in other settings and countries. Such investigations would shed light on the factors impairing or promoting the effectiveness of family nursing. Furthermore, we encourage family nursing researchers to include new predictors of patients’ and family members’ outcomes in their studies (i.e., spirituality of the patients, nurses’ adaptive performance). We also recommend considering several types of outcomes at both the individual and family level (i.e., family functioning and families’ perceived support from health care professionals), which can be assessed via ICE EFFQ and ICE FPSQ respectively.

## Notes

1. The reviewers from the funding agency insisted on including a second comparison group for family relatives, an outpatient group from centers carried out by physicians and physician assistant staff. However, due to an ambulant care type only one measurement occasion was available for this group. Thus, contrary to the inpatient groups, accounting for admission values of the outcome variable was not possible. For the sake of completeness and transparency, we report those additional results. There were no differences between the three groups (inpatient intervention group, inpatient control group, and outpatient control group) with respect to stress after accounting for year of birth (ANCOVA), *F*(2, 186) = 1.14, *p* = .322, $\eta_{p}^{2}$ = .01. Similarly, ANOVA results indicate that there were no differences with respect to anxiety, *F*(2, 187) = 1.96, *p* = .144, $\eta_{p}^{2}$ = .02, and physical symptoms, *F*(2, 187) = 0.24, *p* = .790, $\eta_{p}^{2}$ = .00. Finally, the satisfaction with care differences were negligible, too, according to the ANCOVA results (sex and year of birth as covariates), *F*(2, 124) = 0.46, *p* = .635, $\eta_{p}^{2}$ = .01. In sum, additional analyses corroborate the main findings. Sample characteristics of the outpatient group are presented in the Table 4.
